# AI-Based Detection of Dental Features on CBCT: Dual-Layer Reliability Analysis

**DOI:** 10.3390/diagnostics15243207

**Published:** 2025-12-15

**Authors:** Natalia Kazimierczak, Nora Sultani, Natalia Chwarścianek, Szymon Krzykowski, Zbigniew Serafin, Aleksandra Ciszewska, Wojciech Kazimierczak

**Affiliations:** 1 Kazimierczak Clinic, Dworcowa 13/u6a, 85-009 Bydgoszcz, Poland; 2 Faculty of Medicine, Collegium Medicum, Nicolaus Copernicus University in Torun, Jagiellońska 13-15, 85-067 Bydgoszcz, Poland; 3 Independent Researcher, 85-009 Bydgoszcz, Poland; 4 Faculty of Medicine, Bydgoszcz University of Science and Technology, Kaliskiego 7, 85-796 Bydgoszcz, Poland; 5 Department of Maxillofacial Surgery, Poznan University of Medical Sciences, Przybyszewskiego 49, 60-355 Poznan, Poland

**Keywords:** artificial intelligence, cone-beam computed tomography, diagnostic accuracy, computer-assisted diagnosis, patient outcome assessment

## Abstract

**Background/Objectives:** Artificial intelligence (AI) systems may enhance diagnostic accuracy in cone-beam computed tomography (CBCT) analysis. However, most validations focus on isolated tooth-level tasks rather than clinically meaningful full-mouth assessment outcomes. To evaluate the diagnostic accuracy of a commercial AI platform for detecting dental treatment features on CBCT images at both tooth and full-scan levels. **Methods:** In this retrospective single-center study, 147 CBCT scans (4704 tooth positions) were analyzed. Two experienced readers annotated treatment features (missing teeth, fillings, endodontic treatments, crowns, pontics, orthodontic appliances, implants), and consensus served as the reference. Anonymized datasets were processed by a cloud-based AI system (Diagnocat Inc., San Francisco, CA, USA). Diagnostic metrics—sensitivity, specificity, positive predictive value (PPV), negative predictive value (NPV), and F1-score—were calculated with 95% patient-clustered bootstrap confidence intervals. A “Perfect Agreement” criterion defined full-scan level success as an entirely error-free full-mouth report. **Results:** Tooth-level AI performance was excellent, with accuracy exceeding 99% for most categories. Sensitivity was highest for missing teeth (99.3%) and endodontic treatments (99.0%). Specificity and NPV exceeded 98.5% and 99.7%, respectively. Full-scan level Perfect Agreement was achieved in 82.3% (95% CI: 76.2–88.4%), with errors concentrated in teeth presenting multiple co-existing findings. **Conclusions:** The evaluated AI platform demonstrates near-perfect accuracy in detecting isolated dental features but moderate reliability in generating complete full-mouth reports. It functions best as an assistive diagnostic tool, not as an autonomous system.

## 1. Introduction

The widespread adoption of cone-beam computed tomography (CBCT) has fundamentally reshaped modern digital dentistry, providing clinicians with unprecedented three-dimensional (3D) diagnostic capabilities. Its superb spatial resolution and capacity for detailed, multiplanar visualization of oral and maxillofacial structures have made it an indispensable tool across a broad spectrum of dental specializations, including implantology, endodontics, orthodontics, and oral surgery [1,2,3,4]. Accurate identification of existing dental features—such as restorations, root canal treatments, crowns, and implants—is a foundational element of proper documentation, diagnosis, treatment planning, and forensic investigation [5].

However, the clinical advantages of CBCT are accompanied by significant interpretative challenges. The analysis of large, complex volumetric datasets is a time-consuming and cognitively demanding task that requires specialized training. More critically, the interpretation of these images is subject to considerable inter- and intra-observer variability, a well-documented phenomenon that persists even among experienced practitioners and can affect diagnostic reliability [6,7,8]. This inherent variability in human interpretation has created a clear impetus for technological solutions that can enhance diagnostic consistency and workflow efficiency. Medical imaging datasets are inherently imbalanced, with negative findings overwhelmingly outnumbering positive cases. In such conditions, traditional metrics such as accuracy may be spuriously inflated. Moreover, reliance on isolated feature-level metrics ignores the cumulative nature of diagnostic decision-making, where a single missed feature may invalidate the entire clinical interpretation. This represents a critical validation–utility gap.

In response to this challenge, artificial intelligence (AI), particularly in the form of deep learning models such as convolutional neural networks (CNNs), has emerged as a transformative technology in dentomaxillofacial radiology [9,10]. These systems have demonstrated remarkable potential in automating key radiological tasks, including image classification, segmentation, and detection [6,9]. The existing body of literature, including numerous systematic reviews and meta-analyses, has largely validated the high performance of AI on discrete, isolated diagnostic tasks using CBCT data. For instance, meta-analyses report high pooled diagnostic accuracy (approximately 93%) and recall (approximately 91–93%) for various detection and segmentation objectives [6,7]. Commercially available platforms now leverage these capabilities to generate automated dental charts and structured reports, aiming to streamline clinical workflows and provide robust decision support [8,11].

While these atomistic, or feature-level, performance metrics are statistically impressive, they may not accurately reflect the technology’s true utility and safety in a real-world clinical setting. This discrepancy highlights a critical “validation-utility gap”, where high performance on isolated tasks can be misleading [12]. A clinician’s diagnosis is a holistic synthesis of all findings; it is not an average of individual successes but a comprehensive assessment of the entire full-scan case [13]. An AI system that correctly identifies 99% of findings but misses a single critical pathology or feature in a full-mouth assessment is, for that specific patient, 0% effective, as this one error can invalidate an entire treatment plan. This limitation is compounded by the fact that common metrics like accuracy can be deceptive in medical datasets, which are often highly imbalanced, and a high recall score can mask an unacceptably high rate of false positives [14]. From a clinical standpoint, the unit of decision-making is the entire CBCT examination. Therefore, full-scan level performance constitutes the most stringent and clinically relevant endpoint for evaluating the readiness of AI systems for autonomous use. Therefore, there is a pressing need to evolve the validation paradigm for dental AI beyond simple feature-level metrics toward more clinically meaningful, patient-centered endpoints [15].

The aim of this study is to conduct a rigorous, dual-level evaluation of a commercial AI platform’s accuracy in analyzing CBCT scans. We assess its performance first at the traditional tooth level for detecting common dental features and then, critically, at the full-scan level to determine the rate of perfect, error-free diagnostic agreement. This approach seeks to provide a more stringent and clinically relevant measure of the AI’s readiness for autonomous integration into routine dental practice.

## 2. Materials and Methods

### 2.1. Study Design

This retrospective, single-center detection accuracy study was conducted in accordance with the principles of the Declaration of Helsinki. The reporting of the study adheres to the Standards for Reporting of Diagnostic Accuracy Studies (STARD) 2015 guidelines [16]. The study protocol received formal approval from the Bioethics Committee of Collegium Medicum NCU in Torun, Poland for research involving human participants (protocol no.: KB 274/2025, date of approval 23 April 2025). Due to the retrospective and fully anonymized nature of the data, the requirement for individual patient informed consent was waived by the institutional review board. The study protocol is available upon reasonable request from the corresponding author.

### 2.2. Participant Selection

A consecutive series of patients who underwent CBCT imaging at a single private dental center between January 2024 and April 2025 were considered for inclusion. The initial sample consisted of 167 patients. Eligibility was based on the following criteria:

- Inclusion criterion: A standardized large field-of-view of 10 × 13 cm was required to ensure full dentition coverage.
- Exclusion criteria: (1) Presence of mixed dentition (both primary and permanent teeth). Patients with mixed dentition were excluded because the coexistence of primary and permanent teeth can confound automated tooth numbering. (2) CBCT scans with severe artifacts or overall poor image quality that would preclude a reliable diagnostic assessment.

Potentially eligible participants were identified retrospectively through consecutive review of all CBCT scans performed in the clinic’s radiology database between January 2024 and April 2025. This ensured a consecutive series without selection based on clinical indication or outcomes. Following the eligibility criteria, 20 patients were excluded due to the presence of mixed dentition, resulting in a final study cohort of 147 patients.

### 2.3. Image Acquisition

All CBCT images were acquired using a single Hyperion X9 PRO system (MyRay, Imola, Italy) under a standardized protocol (90 kV, 36 mAs, CTDIvol 4.09 mGy, 13 cm FOV, voxel 0.3 mm).

### 2.4. AI Evaluation

Anonymized DICOM datasets were uploaded to a commercial cloud-based platform Diagnocat version 1.0 (Diagnocat Inc., San Francisco, CA, USA; https://diagnocat.com) and processed without access to clinical data. All the datasets were uploaded and AI-evaluated in 8–18 April 2025 to fix the software state for reproducibility.

The software outputs a structured dental chart indexed by FDI tooth number for seven predefined features: Missing tooth (M), Filling (F), Endodontic treatment (E), Pontic (P), Orthodontic appliance (O), Crown (C), and Implant (I). For each tooth position, presence/absence of each feature was recorded as a binary result.

### 2.5. Human Evaluation

One board-certified radiologist and one orthodontist (both with >10 years CBCT reading experience) independently reviewed all scans on a medical-grade diagnostic display in a controlled environment (Barco Coronis Fusion 4MP; syngo.via client v8.9). The Readers were blinded to AI outputs and all patient identifiers. The same seven features were annotated per tooth using FDI numbering. All seven features were defined as binary outcomes (present/absent) based on their radiographic appearance. After the independent reading sessions, the readers jointly assessed the results of the sessions and resolved any disagreements. The consensus of the Reader served later as the reference standard.

### 2.6. Statistical Analysis

#### 2.6.1. Data Handling and Preparation

Data from both the AI platform and the reference standard were compiled into a master database. A data cleaning protocol was applied to harmonize annotations; specifically, multi-label entries were standardized to use a single comma separator without spaces. Blank cells in the raw data, indicating the absence of a finding, were interpreted as true negatives for all features. The unit of analysis was the tooth position, with a total of 4704 positions (147 patients × 32 teeth) evaluated.

#### 2.6.2. Tooth-Level Diagnostic Accuracy Analysis

The diagnostic performance of the AI was evaluated for each of the seven features independently. For each feature, a 2 × 2 contingency table was constructed to classify the AI’s findings (positive/negative) against the expert consensus reference standard. From these tables, the following metrics were calculated: sensitivity, specificity, positive predictive value (PPV), and negative predictive value (NPV). All performance metrics are reported with 95% confidence intervals (CI), calculated using the non-parametric bootstrap method with 3000 replicates. Analyses of variability by tooth complexity (single vs. multiple findings) were exploratory and not pre-specified.

#### 2.6.3. Full-Scan Level Reliability Analysis

To assess the AI’s holistic performance, the entire diagnostic report for each patient was treated as a single unit of analysis. The primary outcome for this analysis was “Perfect Agreement”, a stringent measure defined as a complete, error-free match between the AI report and the reference standard for all 32 tooth positions within a single full-mouth assessment. The proportion of patients achieving Perfect Agreement was calculated with a 95% CI using the Wilson score method.

#### 2.6.4. Sample Size Determination

The intended sample size was pragmatic, including all eligible CBCTs over the 16-month period. Post hoc Buderer precision analysis confirmed adequacy for common features but indicated limited power for rare findings (implants, pontics) [17]. The target was a two-sided 95% confidence interval (CI) with an overall width of approximately 10%. This approach defines the sample size needed to attain the specified precision. The calculations revealed that the existing sample was adequate for most of the observed features except pontics and implants. Consequently, estimates for rarer features should be interpreted cautiously, acknowledging their wider CIs and reduced precision.

The analyses were performed in R 4.5.1 (R Foundation for Statistical Computing) using pROC and caret.

## 3. Results

### 3.1. Patient Characteristics

**Figure 1:**
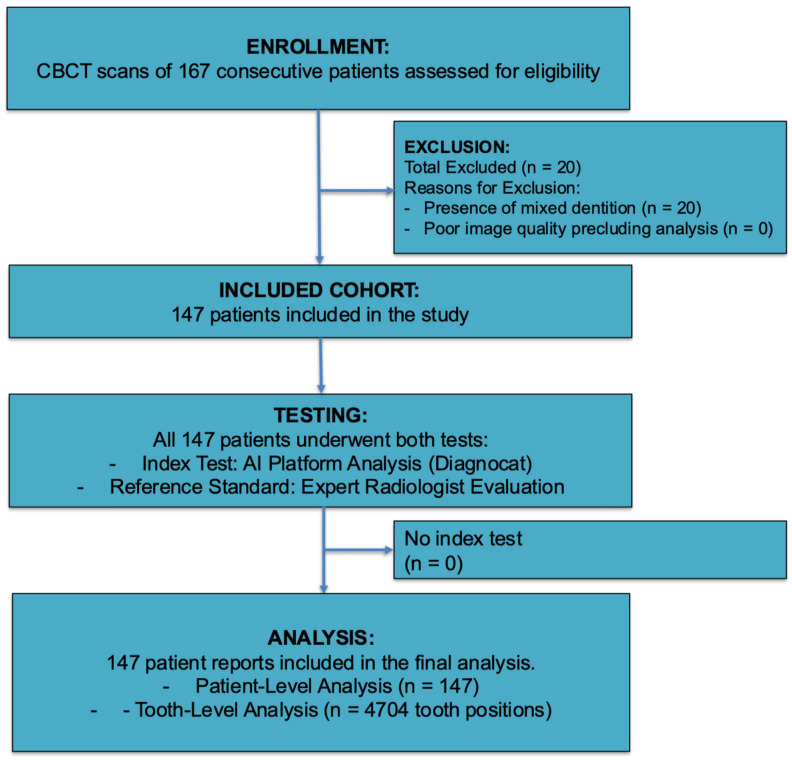
STARD flow diagram of study material.

From an initial pool of 167 consecutive patients, 147 met the inclusion criteria and were included in the final analysis. The participant flow is detailed in the STARD diagram (Figure 1). The cohort had a mean age of 34.5 ± 13.3 years (range: 14–67 years) and was predominantly female (66.0%, *n* = 97). The analysis encompassed a total of 4704 tooth positions. The prevalence of the evaluated features across all tooth positions varied widely, from common findings like Fillings (18.0%) and Missing teeth (14.8%) to rare features such as Pontics (0.7%) and Implants (0.5%).

There were no missing data for index or reference test outcomes after initial exclusions. Due to retrospective nature of the study, no adverse events were recorded. No indeterminate results were encountered.

### 3.2. Tooth-Level Diagnostic Accuracy

**Figure 2:**
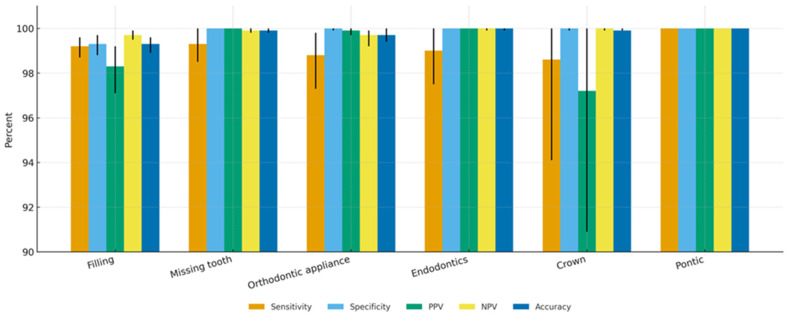
Tooth-level detection accuracy metrics for the AI software across seven dental features, including missing teeth, fillings, endodontically treated teeth, pontics, orthodontic appliances, crowns. Bars represent point estimates for sensitivity, specificity, positive predictive value (PPV), negative predictive value (NPV), accuracy, and Cohen’s kappa. Error bars indicate 95% confidence intervals. The Y-axis is scaled from 75% to enhance visualization of differences between metrics.

AI achieved outstanding diagnostic accuracy across all features (Table 1, Figure 2). Sensitivity ranged from 98.6% for crowns to 100% for implants and pontics, while specificity exceeded 99.3% for all categories. PPV and NPV were consistently high (≥97.2% and ≥99.7%, respectively), and F1-scores exceeded 97.9% for all non-rare features. Implants and pontics achieved perfect estimates (100% across all metrics), although the small number of positive cases warrants cautious interpretation.

**Figure 3:**
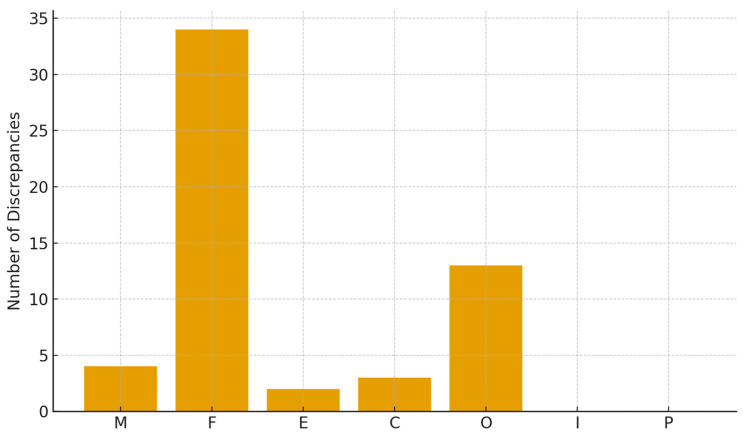
Distribution of discrepancies (M = missing tooth; F = filling; E = endodontically treated tooth; C = crown; O = orthodontic appliance; I = implant; P = pontics).

A total of 56 tooth-level discrepancies were identified across the dataset (Figure 3). The majority arose from two categories:

- Fillings: 34 discrepancies (60.7%);
- Orthodontic appliances: 13 discrepancies (23.2%).

Other features accounted for small proportions:

- Missing teeth: 4 (7.1%);
- Crowns: 3 (5.4%);
- Endodontics: 2 (3.6%).

**Figure 4:**
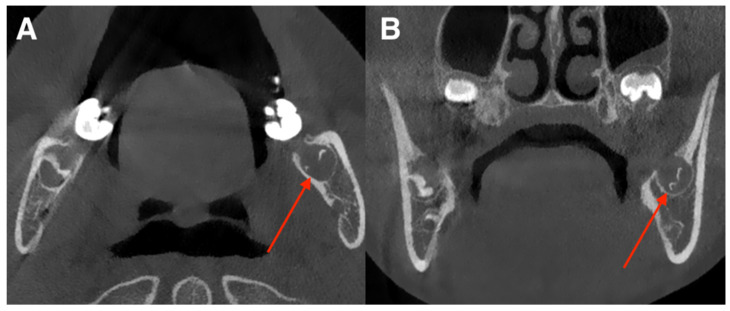
Example of a false-positive “missing tooth” detection for tooth 38. The red arrows indicate the developing germ of tooth 38 in a 15-year-old male. (**A**) Axial view. (**B**) Coronal view. Tooth 48 was correctly annotated as present.

Pontics and implants showed no discrepancies. Most discrepancies consisted of false positives in fillings (*n* = 23) or false negatives in orthodontic appliances (*n* = 12). Sample false positive missing tooth classification is presented in Figure 4.

### 3.3. Anatomical Distribution of Errors

Errors were not uniformly distributed across the dentition and have been clustered predominantly in the posterior teeth, particularly mandibular molars:

- Tooth 36: 6 errors (4.1% of patients);
- Tooth 27: 3 errors;
- Tooth 21, 11, 17, 14, 46, 38: isolated errors (1–2 cases).

Anterior teeth demonstrated very low error rates, frequently 0%.

This pattern mirrors known challenges in CBCT interpretation, where multi-rooted posterior teeth exhibit greater anatomical complexity, metallic artifacts, and overlapping structures.

A logistic model comparing molar teeth with premolars and anterior teeth showed Odds Ratio (OR) = 1.76 (95% CI: 0.96–3.20), indicating a trend toward higher error risk in molars, consistent with the anatomical distribution analysis and prior PAN findings.

### 3.4. Full-Scan Level Diagnostic Accuracy

**Figure 5:**
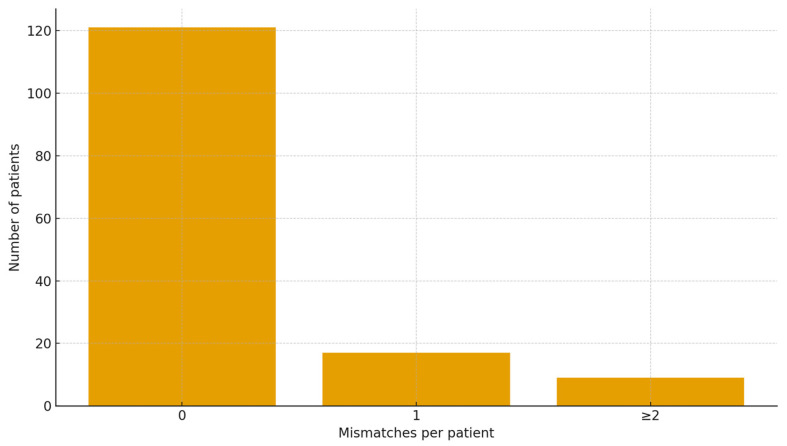
Histogram showing the distribution of discrepancies per full CBCT scan. The bar chart illustrates the number of patients achieving Perfect Agreement (0 mismatches) versus those with one or more diagnostic errors.

However, when the analytical perspective shifted from the individual tooth to the holistic full-mouth assessment—a perspective that more closely mirrors clinical reality—the AI’s reliability decreased substantially. A “Perfect Agreement” was defined as a complete, error-free match between the AI report and the expert reference standard for a full-mouth assessment. Perfect Agreement was achieved in 82.3% of patients (75.8–87.8%, 95% CI). Thus, while per-tooth and per-feature full-scan level metrics are near-perfect, approximately 18% of patients still had at least one discrepancy somewhere in the full-mouth report. Of total number of 147 patients, 26 (17.7%) had ≥1 discrepancy (17 patients with a single error; 9 with ≥2 errors). The data are presented in Table 2 and Figure 5.

At the tooth level, the overall mismatch rate was low and varied modestly by tooth group (molars 1.14%, incisors 1.02%, premolars 0.60%, canines 0.34%).

Omissions had tendency to cluster in teeth within multiple reader-identified findings:

- False negative rate rose from 0.31 to 0.85% on single-finding teeth to ~2–3% on multi-finding teeth, depending on feature (e.g., Fillings 0.31% → 2.29%; Orthodontic appliances: 0.85% → 2.45%; Crowns: 0.00% → 2.78%; Endodontic treatment: 0.00% → 1.02%).
- Taxonomically, we observed 14 “omission on complex tooth” cases vs. 12 “omission on simple tooth”.
- The most frequent complex combo was Filling + Orthodontic appliance (*n* = 181 teeth); within these, the AI most often missed the filling (8 omissions) more than the appliance (3 omissions). For Endodontic treatment + Filling (*n* = 153), omissions were rare (1 endo miss). For Crown + Endodontic treatment (*n* = 21), sporadic misses occurred (1 endo, 1 crown).

**Figure 6:**
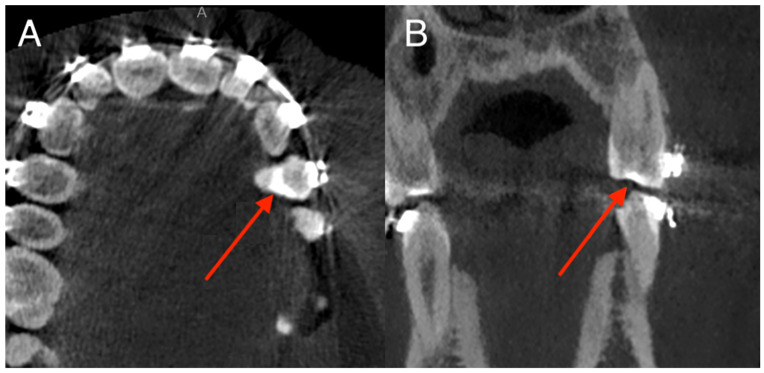
Example of a false-negative filling diagnosis for tooth 24. Red arrows indicate a composite filling on tooth 24, which also carries an orthodontic appliance in an 18-year-old female. (**A**) Axial view. (**B**) Coronal view.

One of the typical cases of omissions (false negative diagnosis of dental filling in tooth with orthodontic appliance) is presented in Figure 6.

## 4. Discussion

This study provides a dual-perspective evaluation of a commercial AI platform for CBCT analysis, revealing a significant paradox: the system demonstrates excellent accuracy at the micro-level of individual teeth but exhibits low reliability at the macro-level of the whole patient. While the tooth-level performance is consistent with, and in some cases superior to, findings from previous studies [9,18,19], the full-scan level analysis presents a starkly different picture that questions the platform’s readiness for autonomous clinical use.

The central finding of this study is the dramatic drop from near-perfect tooth-level accuracy (over 99% for most features) to a more modest 82.3% rate of perfect, error-free agreement at the full-scan level. This means that for nearly one in five patients, the automated report contained at least one error. Our analysis reveals that this discrepancy is not random but is systematically linked to clinical complexity. The AI’s performance faltered specifically on teeth with multiple co-existing findings (e.g., a filling on an endodontically treated tooth or a crown adjacent to an orthodontic appliance). The false negative rate increased substantially on “complex” teeth presenting multiple co-existing findings compared to teeth with single findings. This suggests the algorithm may be adept at identifying the most prominent feature but struggles with the cognitive task of identifying secondary or tertiary findings, a challenge analogous to ‘satisfaction of search’ in human radiological interpretation.

These findings have profound implications for the validation paradigm of clinical AI in dentistry. The current body of literature is dominated by studies reporting atomistic metrics for isolated tasks. As our results demonstrate, tooth-level metrics can create a misleading impression of real-world utility, especially in imaging of the whole orofacial area as panoramic radiography or large FOV CBCT. A clinician’s diagnosis is a holistic synthesis; a single critical error of omission can invalidate an entire treatment plan or severely compromise patients’ health. Such error can render an otherwise “99% accurate” report a 100% failure for that specific patient. This highlights a critical “validation-utility gap”, where impressive statistical performance on isolated tasks does not translate to perfect clinical reliability [13,20]. Therefore full-scan level agreement should be considered a primary endpoint in future AI-validation studies. Therefore, the paired reporting both on l feature-level and full-scan level in validation studies is mandatory in diagnostic accuracy studies [16]. Given the importance of full-scan level performance in AI validation, particularly for commercially available tools, we regard this level as paramount from the standpoint of patient safety. This shift toward more holistic evaluation is becoming an established practice in other medical imaging domains, where performance is often reported at both the feature-level and full-scan level to provide a more complete picture of clinical readiness [21,22,23,24].

The current body of evidence on the clinical utility of Diagnocat in CBCT evaluation has primarily focused on granular, tooth-level assessments (Table 3) [18,19,25,26,27]. Kazimierczak et al. [19] reported strong performance in evaluating endodontic treatment outcomes on CBCT scans, with high accuracy for detecting features such as filling quality, overfilling, voids, and root canal anatomy, often exceeding 90%. In a recent study, Diagnocat showed high agreement (89%) with experienced endodontists in detecting apical radiolucencies, although clinicians still outperformed the system in sensitivity and overall accuracy. The tool performed particularly well in identifying healthy cases, indicating high specificity and utility for ruling out disease [26]. However, its accuracy in this task depends heavily on imaging modality, with significantly better performance on CBCT than on panoramic images [26]. Ezhov et al. found that Diagnocat significantly improved dentists’ diagnostic sensitivity and specificity when interpreting CBCT scans, with aided dentists outperforming unaided counterparts [25]. The authors evaluated the program’s sensitivity and specificity for detecting multiple dental conditions, including indicators of prior dental treatment. Overall performance on these tasks was high, although the diagnostic accuracy metrics were slightly lower than those observed in our study. Reported sensitivities and specificities for detection of artificial crowns were 0.9546 and 0.9963, respectively; for endodontically treated teeth, 0.9676 and 0.9953; for fillings, 0.9721 and 0.9921; for implants, 0.9727 and 0.9997; for missing teeth, 0.9824 and 0.9405; and for pontics, 0.9101 and 0.9998. The modest tooth-level improvements observed in our results may be attributable to enhancements in the program’s algorithms implemented in the interval between the Ezhov’s study and ours.

Based on this dual-level analysis, the evaluated AI platform’s optimal role is that of a powerful assistive technology or “second reader”. The platform’s exceptionally high specificity and negative predictive value across all features give clinicians a high degree of confidence in its negative findings; if the AI reports a feature is absent, it is very likely absent. The evidence supports the role of Diagnocat as a tool for augmenting human performance. Its primary value in CBCT assessment, as demonstrated by this study, is its ability to mitigate the risk of diagnostic error, particularly errors of omission. This aligns with the well-documented challenges in radiology, where human factors such as fatigue, satisfaction of search (the tendency to stop searching for abnormalities after one is found), and other cognitive biases can lead to missed findings [28]. However, the clinician must remain the ultimate arbiter of the final diagnosis, particularly in complex cases and in patients with compromised dental status. Clinicians should apply heightened scrutiny to clinically complex teeth and to regions where the priori risk of error is highest.

The study has a few limitations. First, the retrospective, single-center design may limit the generalizability of our findings to other patient populations or imaging hardware. Second, our reference standard was based on the consensus of two experienced specialists. While a robust method, a larger multi-reader panel would represent a more definitive gold standard, especially given the known challenges of inter-observer variability in radiology. Third, our post hoc power analysis confirmed that while the sample size was adequate for common features, the study was underpowered for the rarest findings (implants and pontics). Consequently, the perfect performance metrics observed for these features should be interpreted with caution due to their wider confidence intervals. Finally, as with many studies of commercial software, the proprietary “black box” nature of the algorithm prevents a deeper analysis of the specific architectural reasons for its failure modes. Future multi-center, prospective studies are needed to validate these findings and further explore the impact of such tools on clinical decision-making and patient outcomes.

## 5. Conclusions

The Diagnocat AI platform demonstrates excellent accuracy in detecting isolated dental features on CBCT scans but achieves perfect full-scan diagnostic agreement in only 82.3% of patients. Performance limitations are driven primarily by omission errors in clinically complex teeth. While the system is not currently suitable for autonomous clinical deployment, it represents a valuable assistive tool that can enhance workflow efficiency and diagnostic safety.

Future prospective multi-center studies should validate these findings across diverse scanners and patient populations. Direct head-to-head comparison of multiple commercial AI platforms against expert consensus reference standards is also warranted to determine comparative effectiveness.

## Figures and Tables

**Table 1:** Tooth-level diagnostic accuracy with patient-clustered 95% CIs (3000 replicates).

Feature	Sensitivity	Specificity	PPV	NPV	Accuracy	F1
Missing tooth	99.3% (98.5–100.0%)	100.0% (100.0–100.0%)	100.0% (100.0–100.0%)	99.9% (99.8–100.0%)	99.9% (99.8–100.0%)	99.7% (99.2–100.0%)
Filling	99.2% (98.7–99.6%)	99.3% (98.8–99.7%)	98.3% (97.1–99.2%)	99.7% (99.5–99.9%)	99.3% (98.9–99.6%)	98.7% (98.0–99.3%)
Endodontically treated tooth	99.0% (97.5–100.0%)	100.0% (100.0–100.0%)	100.0% (100.0–100.0%)	100.0% (99.9–100.0%)	100.0% (99.9–100.0%)	99.5% (98.7–100.0%)
Pontic	100.0% (100.0–100.0%)	100.0% (100.0–100.0%)	100.0% (100.0–100.0%)	100.0% (100.0–100.0%)	100.0% (100.0–100.0%)	100.0% (100.0–100.0%)
Orthodontic appliance	98.8% (97.3–99.8%)	100.0% (99.9–100.0%)	99.9% (99.7–100.0%)	99.7% (99.2–99.9%)	99.7% (99.4–100.0%)	99.4% (98.6–99.9%)
Crown	98.6% (94.1–100.0%)	100.0% (99.9–100.0%)	97.2% (90.9–100.0%)	100.0% (99.9–100.0%)	99.9% (99.9–100.0%)	97.9% (93.7–100.0%)
Implant	100.0% (100.0–100.0%)	100.0% (100.0–100.0%)	100.0% (100.0–100.0%)	100.0% (100.0–100.0%)	100.0% (100.0–100.0%)	100.0% (100.0–100.0%)

**Table 2:** Distribution of errors in patients.

Criterion	Percent of Patients	95% CI
0 errors (Perfect Agreement)	82.3%	75.8–87.8%
≤1 error	91.8%	87.1–96.0%
≤2 errors	94.6%	90.5–98.0%

**Table 3:** Comparison of current results with recent literature.

Study	Year	Modality	Feature	Metric
Current Study	2025	CBCT	Fillings	Sens: 99.2%/Spec: 99.3%
Ezhov et al. [25]	2021	CBCT	Fillings	Sens: 97.2%/Spec: 99.2%
Kazimierczak [19]	2024	CBCT	Endodontics	Accuracy: >90%
Allihaibi [26]	2025	CBCT	Periapical	Agreement: 89%

## Data Availability

The raw data supporting the conclusions of this article contain patient diagnostic information and are therefore subject to privacy and legal restrictions. The data are available from the authors upon reasonable request.

## Abbreviations

AI	Artificial Intelligence
CBCT	Cone-Beam Computed Tomography
PPV	Positive Predictive Value
NPV	Negative Predictive Value
CI	Confidence Interval
STARD	Standards for Reporting of Diagnostic Accuracy Studies
OR	Odds Ratio
OPG	Orthopantomogram
MRI	Magnetic Resonance Imaging
CNN	Convolutional Neural Network
FOV	Field of View
PAN	Panoramic Radiography
